# Factors Influencing Nursing Internship Students’ Readiness to Use AI: Cross-Sectional Study Using Neural Network Analysis

**DOI:** 10.2196/92533

**Published:** 2026-08-18

**Authors:** Sameer A Alkubati, Wesam T Almagharbeh, Talal A Alqalah, Basma Salameh, Hamdan Albaqawi, Awatif M Alrasheeday, Abdulhafith Alharbi, Layla Alshammari, Bushra Alshammari, Eddieson Pasay-an, Anwar Abdulkareem, Shimmaa Mohammed Elsayed

**Affiliations:** 1 Department of Medical Surgical Nursing, College of Nursing, University of Ha'il, Hail University, Hail, Ha'il Region, 00966, Saudi Arabia, 966 506575284; 2 Medical Surgical Nursing Department, University of Tabuk, Tabuk, Tabuk Region, Saudi Arabia; 3 Department of Nursing, Arab American University, Jenin, Palestinian Territory; 4 Nursing Administration Department, Faculty of Nursing, University of Hail, Hail, Saudi Arabia; 5 Department of Psychiatric and Mental Health Nursing, College of Nursing, University of Hail, Hail, Saudi Arabia; 6 Fundamental of Nursing Department, College of Nursing, King Khalid University, Abha, Saudi Arabia; 7 Artificial Intelligence and Data Science Department, College of Computer Science and Engineering, College of Computer Science and Engineering, University of Hail, Hail, Saudi Arabia; 8 Critical Care and Emergency Nursing Department, Faculty of Nursing, Damanhour University, El Beheira, Egypt

**Keywords:** nursing internship students, attitude, perception, self-efficacy, barriers, anxiety, readiness, AI

## Abstract

**Background:**

Enhancing nursing students’ awareness, attitudes, beliefs, and preparedness toward AI may help improve their health care knowledge and practice.

**Objective:**

This study aimed to assess nursing students’ attitudes, perceptions, self-efficacy, barriers, and anxiety, which influence their readiness to adopt AI in nursing practice.

**Methods:**

This study used a cross-sectional, correlational design. Data were collected from 307 nursing internship students using an 8-part, self-administered questionnaire.

**Results:**

Increased self-efficacy with computers was correlated with decreased barriers to accessing AI technology, lower computer anxiety scale scores (*r*=−0.27, *P*<.001 and *r*=−0.57, *P*<.001, respectively), and higher perceptions of using AI (*r*=0.27, *P*<.001). Meanwhile, nursing students’ readiness to adopt AI in nursing practice was negatively associated with barriers to accessing AI technology (*r*=−0.20, *P*<.001) and positively associated with attitudes toward and perceptions of using AI (*r*=0.32, *P*<.001 and *r*=0.14, *P*=.01, respectively). Increased barriers to accessing AI technology were associated with negative attitudes toward AI and nursing students’ perceptions of using AI (*r*=−0.34, *P*<.001 and *r*=−0.39, *P*<.001, respectively). A multilayer neural network model identified barriers (relative importance=0.27), attitudes (relative importance=0.16), and perceptions (relative importance=0.15) as the most significant predictors, while self-efficacy (relative importance=0.11) and anxiety (relative importance=0.07) showed smaller contributions, despite nonsignificant bivariate associations with nursing students’ AI readiness. The model demonstrated strong predictive performance, achieving a low relative error of 0.62 in the training set. The stability and generalization ability of the model were supported by the training and testing set results, which yielded a training sum of squares error of 65.93 and a testing sum of squares error of 35.49, showing no signs of overfitting.

**Conclusions:**

Several contributing factors influenced nursing students’ readiness to embrace AI, with barriers, attitudes, and perceptions emerging as the most consistent, whereas self-efficacy and anxiety may play indirect roles. To improve the adoption of AI among nursing students, such factors should be dealt with in such educational programs; an interrelated adoption of AI in nursing practice is expounded as a more favorable environment.

## Introduction

AI refers to a broad category of technology that empowers the hardware and software of computers to emulate the intelligent behavior of humans and perform at a level comparable to that of humans [1,2]. AI significantly enhances nursing care procedures, improves patient outcomes, and increases the effectiveness of care delivery [3,4]. It can enhance clinical decision-making by offering nurses insightful data and recommendations supported by evidence to inform patient care decisions [5]. AI can help nurses diagnose and treat patients more accurately by analyzing large amounts of data [6]. It can also support predictive analytics, improve patient monitoring, and help nurses identify the early warning signs of deterioration [3]. It is becoming more important for nursing education programs to prepare student nurses with the skills they need to use AI technology and to embrace its use in nursing practice as it develops [7-9]. The health care system requires this level of training in light of technological improvements and guarantees that nurses can use AI for patient benefits [8,10]. Enhancing nursing students’ awareness, attitudes, beliefs, and preparedness toward AI may help improve their care [11-13]. To determine whether nursing students are prepared to incorporate AI-based technology into their academic work, it is important to examine various factors including their attitudes, perceptions, anxiety, self-efficacy, and barriers to the value of AI. Thus, it is critical to train nursing students to efficiently use AI technology and provide innovative health care delivery. In addition, nursing students’ competencies need to assess their self-efficacy in using computers and their AI literacy to optimize the use of AI sources [14].

Although there has been an increase in the involvement of AI in nursing practice and education, researchers have focused on investigating nursing students’ interest and readiness to adopt AI in the future [15,16]. One study revealed that nursing students’ intention to use AI in the health care system can be affected by their positive attitudes and self-efficacy [17]. According to a study by Labrague et al [16], self-rated technological ability, knowledge of AI-powered technology, and perceived usage of AI in nursing practice all influenced Philippine nursing students’ modest preparedness to incorporate AI into their practice. Accessibility issues with technology may also influence intentions and future behaviors [18,19]. These obstacles may be caused by a lack of computer proficiency, lack of understanding of AI, lack of money, or time constraints [20]. This study aimed to assess nursing students’ attitudes, perceptions, self-efficacy, barriers, and anxiety that influence their readiness to adopt AI in nursing practice.

## Methods

### Design

A cross-sectional correlation design was used in this study.

### Setting

This study was conducted among nursing students at the College of Nursing, Damanhour University, from November 2024 to January 2025. This faculty serves nursing students in the Beheira Governorate, the first accredited faculty member at Damanhour University. It consists of 4 years, with 1 year of internship training in hospitals. Students experience technology when dealing with online lectures, quizzes, and assignments. Nursing students are obligated to complete and pass both required and elective courses within a specific time frame to join the internship program. Students in the nursing program spend a year working under the supervision of faculty members in a variety of hospital settings as part of the internship program’s primary goal of preparing them for real-world nursing.

### Study Population and Sampling

The total number of nursing students engaged in the internship program was 1400. The required sample size of 302 internship students was calculated using the OpenEpi online calculator, version 3.01 [21], according to the criteria of 5% absolute precision and a 95% confidence level. A total of 350 internship students were invited to participate, and 307 (response rate: 87.7%) completed the survey. Students completing nursing internships with a minimum of 2 months of clinical experience were eligible to participate.

### Tools of the Study

Data were collected using 8 sections of a self-administered questionnaire. The first section included the participants’ sociodemographic details, including age, sex, level of education, previous year’s grade, duration of internet use, awareness of AI in nursing practice and health care, previous AI knowledge, and sources of information. The second section assessed the technological abilities of nursing students, as adopted from Labrague et al [16], using a single question: “In general, how do you assess your technological proficiency?” to gauge the technological abilities of nursing students. On a 5-point Likert scale, which goes from “1=basic knowledge” to “5=expert,” participants scored their level of technology competency; higher scores denote greater technological skill. As this is a single-item measure, the original study established the reliability of the instrument using test-retest reliability (*r*=0.910) [16]. Likewise, this study demonstrated excellent test-retest reliability (*r*=0.902), consistent with recommendations that test-retest reliability is an appropriate method for evaluating the reliability of single-item measures [22,23].

The third section, developed by Knezek [24], assessed nursing students’ self-efficacy using computers. It consists of 8 self-reported questions, with a 4-point Likert scale ranging from 0 (strongly disagree) to 3 (strongly agree), and 6 items that are reverse-scored. The total rating ranges from 0 to 24, with higher scores indicating better computer self-efficacy. Although this scale was originally developed prior to the emergence of contemporary AI technologies, it assesses general computer self-efficacy, which is considered a foundational determinant of an individual’s ability to engage with and adapt to new digital tools including AI-based systems. The questionnaire was tested for reliability with a Cronbach α of 0.801 [24]. In this study, the reliability of the scale was confirmed by Cronbach α of 0.875.

The fourth part assessed nursing students’ readiness to adopt AI using a 5-item scale originally developed by Ayanwale et al [25]. In this study, the scale was adopted based on its prior modification and application among nursing students by Labrague et al [16]. To enhance its relevance to the nursing education context, Labrague et al [16] modified the original teacher-oriented items by replacing the term “teach” with “learn,” “class” with “nursing practice,” and “teaching in class” with “nursing curriculum.” These contextual modifications ensured that the scale more accurately reflected AI readiness among nursing students, while preserving the conceptual framework of the original instrument [16]. It consists of 5 self-reported questions to assess nursing students’ readiness to adopt AI in nursing practice. Each question is scored on a 5-point Likert scale ranging from “strongly disagree” (1) to “strongly agree” (5). Higher scores indicate greater willingness to use AI-based technologies. We divided this number by the number of items and added the average score for each item to determine the final score. Internal consistency was assessed using Cronbach α, which yielded a value of 0.920. The reliability of the scale was confirmed using Cronbach α, which was 0.937.

The fifth part assessed students’ perceptions of the use of AI using an 11-item scale developed by Swan [26]. Participants used a 5-point Likert scale, with 1 denoting “strongly disagree” and 5 denoting “strongly agree,” to score their level of agreement. The implementation and use of AI technology in nursing practice were perceived more favorably by those who scored higher on this scale. The reliability of the scale was established with a Cronbach α of 0.900 [26]. In this study, the reliability of the scale was confirmed by Cronbach α, which was 0.836.

The sixth part, adopted from Ayanwale et al [25], was used to assess nursing students’ perceived barriers to using AI technology. Each item is scored on a 5-point Likert scale ranging from “strongly disagree” (1) to “strongly agree” (5). Higher ratings suggested that participants felt there were more obstacles to using the AI-powered solutions. The internal consistency of the scale, as determined by Cronbach α, was 0.880 [25]. In this study, the reliability of the scale was confirmed by Cronbach α, which was 0.894.

The seventh part assessed nursing students’ attitudes toward AI, which was developed by Schepman and Rodway [27]. The questionnaire consisted of 20 self-report questions on a 5-point Likert scale, with a higher score indicating a positive attitude toward AI [27]. The reliability of the scale was established with a Cronbach α of 0.930 [28]. In this study, the reliability of the scale was confirmed by Cronbach α, which was 0.899.

The eighth part was the Short Computer Anxiety Scale, which was adopted from Lester et al [29]. A 6-item measure was developed that included questions on computer confidence. Of these, 2 items showed computing comfort, whereas the other 4 items showed shortcomings. Internal reliability was confirmed by Cronbach α of 0.760 [29]. In this study, the reliability of the scale was confirmed by Cronbach α, which was 0.844. All study instruments were administered in their original English versions, since English is the official language of instruction for internship nursing students. Therefore, no translation procedure was required.

### Ethical Considerations

This study was approved by the Damanhour Research Ethics Committee (Approval 2024‐030685). Following an explanation of the study’s purpose, all the respondents provided written informed consent. The participants were made aware of their freedom to leave the study at any moment and without any explanation. Confidentiality of the respondents’ data and anonymity were guaranteed. During their breaks, all intended nursing students received an invitation to participate in the study, as well as informed consent forms and questionnaires.

### Data Analysis

SPSS statistics (version 26, IBM Corp) was used to evaluate the data, with a significance level of less than .05. While the mean and SD are used to summarize regularly distributed continuous data, frequencies and percentages were used to show categorical data. Histogram and statistical tests (Shapiro-Wilk and Kolmogorov-Smirnov tests) were used to determine whether the data were normal. The data were deemed to be normally distributed because the *P* values of these tests were higher than .05. One-way ANOVA (for more than 2 groups) and 2-tailed independent-sample *t* tests (for 2 groups) were used to examine the association between nursing students’ attributes and overall scores. Pearson correlation coefficients were used to determine the correlations between variables. A multilayer perceptron neural network was used to predict nursing students’ readiness to use AI in nursing practice. The network architecture consisted of 3 interconnected layers: an input layer, a single hidden layer, and an output layer. The input layer comprised categorical demographic factors (automatically dummy-coded into distinct operational units covering age categories, sex, study levels, internet duration, previous semester grade point average [GPA], and technological proficiency levels) and continuous variables (computer self-efficacy, perception, attitude, and computer anxiety). Of the 307 participants, the dataset was randomly partitioned into training (n=215, 70.0%) and testing (n=92, 29.9%) sets to prevent overfitting. The model was trained and its performance was evaluated using the sum of squares error (SSE) and relative error. The choice of a multilayer perceptron neural network was justified by its capacity to describe complicated nonlinear relationships and interactions between factors that typical linear regression models may not sufficiently capture. While regression and structural equation modeling are frequently used and have good interpretability, they often assume linearity and may be limited in capturing higher-order interactions between psychological and environmental variables [30].

## Results

### Participant Characteristics

The sample (N=307) distributions are presented in Table 1. It reveals that the mean age of undergraduate nursing students was 20.0 (SD 1.05) years, with 69.4% (n=213) being 20 years or younger. Most students were female (n=185, 60.3%). Most nursing students (n=143, 46.6%) had an excellent GPA during the semester, and 44.6% (n=137) had a moderate level of technology proficiency. While a clear majority of undergraduate nursing students (n=219, 71.3%) possessed general, baseline knowledge about AI as a broad consumer concept, a substantially smaller proportion (n=126, 41.0%) demonstrated awareness regarding the specific, practical applications of AI within health care systems.

**Table 1.** Demographic characteristics of undergraduate nursing students (N=307).

Demographic characteristics	Values, n (%)
Age (y)	
≤20	213 (69.4)
>20	94 (30.6)
Sex	
Male	122 (39.7)
Female	185 (60.3)
Previous grade	
Poor	5 (1.6)
Fair	15 (4.9)
Good	30 (9.8)
Very good	114 (37.1)
Excellent	143 (46.6)
Technical proficiency	
Bad	5 (1.6)
Accepted	41 (13.4)
Middle	137 (44.6)
Above the average	86 (28.0)
Excellent	38 (12.4)
Awareness of AI in health care (yes/no)	
Yes	126 (41.0)
No	181 (59.0)
Awareness of AI in nursing practice (yes/no)	
Yes	125 (40.7)
No	182 (59.3)
Previous knowledge about AI (yes/no)	
Yes	219 (71.3)
No	88 (28.7)

Students aged over 20 years, studying at the fourth level, and having an excellent GPA in the last semester had a higher mean self-efficacy with computers, with statistically significant *P* values of .04, .04, and .001, respectively. Nursing students’ readiness to adopt AI in nursing practice was higher among male students, those with excellent technological proficiency, those aware of AI use in health care and nursing care, and those with prior knowledge of AI, with statistically significant *P* values of .02, <.001, <.001, <.001, and <.001, respectively.

Nursing students’ perception of AI had a higher mean score among those aware of AI’s use in health care and nursing care, with a statistically significant *P* value of <.001. However, barriers to accessing AI technology were higher among students at the fourth level, with a statistically significant *P* value of .006. Nursing students’ attitudes toward AI were more positive among those with excellent technological proficiency, those aware of AI’s use in health care and nursing care, and those with prior knowledge about AI, with statistically significant *P* values of .04, .03, .008, and <.001, respectively. Finally, computer anxiety was higher among students aged 20 years or younger and those with fair GPAs in the last semester, with statistically significant *P* values of .01 and <.001, respectively. Further details are provided in Table 2.

**Table 2.** Association between demographic characteristics with self-efficacy, readiness, perception, barriers, attitude, and anxiety.

Variables	Self-efficacy	Readiness	Perception	Barriers	Attitude	Anxiety
Age, mean (SD)						
≤20 years	20 (4.0)	15 (4.6)	13.2 (3.7)	14.4 (2.9)	59.2 (8.6)	10.5 (3.4)
>20 years	21.2 (4.8)	14.1 (5.0)	13.8 (3.8)	14.8 (3.1)	59.3 (10.3)	9.4 (3.5)
*P* value	.04	.12	.22	.26	.93	.01
Sex, mean (SD)						
Male	20.5 (4.5)	15.5 (4.8)	13.8 (3.8)	14.7 (2.7)	60.2 (9.1)	10 (3.5)
Female	20.3 (4.1)	14.2 (4.7)	13.1 (3.7)	14.4 (3.1)	58.6 (9.2)	10.2 (3.4)
*P* value	.62	.02	.16	.37	.12	.61
Study level, mean (SD)						
First	19.4 (3.9)	14.8 (4.3)	12.9 (3.8)	14.2 (2.7)	59.5 (7.7)	10.7 (3)
Second	20.8 (4)	15.4 (4.6)	13.7 (3.2)	14.8 (2.9)	59.7 (9.5)	10.3 (3.9)
Third	20.4 (5.2)	13.6 (5)	13 (4.1)	13.8 (3.3)	57.4 (10.7)	9.4 (3.3)
Fourth	21.3 (4)	14.7 (5.2)	14.1 (3.9)	15.5 (3)	60.1 (8.7)	9.8 (3.3)
*P* value	.04	.12	.16	.006	.31	.08
Internet duration (h), mean (SD)						
<3	20 (4.6)	14.2 (5.0)	13 (3.7)	14.2 (3.5)	58.8 (10.5)	10.7 (3.6)
3‐6	20.4 (4)	15.4 (4.4)	13.4 (3.9)	14.5 (2.6)	58.8 (7.6)	10.1 (3.4)
>6	20.7 (4.5)	14.1 (5.0)	13.6 (3.7)	15 (3)	60.2 (10.1)	9.7 (3.4)
*P* value	.57	.08	.60	.25	.52	.12
Last semester GPA^a^, mean (SD)						
Poor	18.8 (6.7)	12 (5.1)	11 (4.9)	16.4 (6.1)	54 (11.3)	8.8 (1.9)
Fair	16.7 (3)	13.5 (3.8)	14 (3.2)	15.4 (2.4)	59.6 (11.3)	14.5 (4.3)
Good	18.9 (4)	15 (4.3)	13.2 (3.3)	13.9 (2.5)	59.9 (8.3)	10.2 (3.2)
Very good	20.4 (4.2)	14.2 (4.9)	13.7 (3.9)	14.4 (2.6)	58.8 (8.4)	10.1 (3.2)
Excellent	21.1 (4.3)	15.2 (4.7)	13.2 (3.8)	14.6 (3.3)	59.6 (9.7)	9.8 (3.4)
*P* value	.001	.23	.50	.31	.68	<.001
Technological proficiency, mean (SD)						
Bad	20 (4.8)	8.2 (3.4)	13 (6.0)	12.4 (2.9)	51.4 (11.8)	11 (2.5)
Accepted	20.5 (4.6)	13.8 (4.7)	14 (4.2)	14.4 (4.1)	58.3 (10.9)	9.8 (3.8)
Middle	19.9 (4.3)	13.7 (4.5)	13.6 (3.8)	14.7 (3)	58.6 (8.6)	10.6 (3.5)
Above the average	20.9 (3.7)	16.3 (4.6)	12.9 (3.2)	14.7 (2.5)	59.7 (8.9)	9.6 (3)
Excellent	20.9 (5.1)	16.5 (4.4)	13.1 (4.0)	14.1 (2.5)	62.6 (8.5)	10 (3.8)
*P* value	.43	<.001	.53	.36	.04	.29
Awareness AI used in health care, mean (SD)						
Yes	20.9 (4.3)	16.4 (4.8)	14 (3.90)	14.3 (2.9)	60.6 (8.2)	10 (3.3)
No	20 (4.3)	13.5 (4.3)	12.5 (3.4)	14.8 (3)	58.2 (9.7)	10.2 (3.6)
*P* value	.09	<.001	<.001	*.*16	.03	.65
Awareness AI used in nursing care, mean (SD)						
Yes	20.8 (4.2)	16.5 (4.8)	14 (3.9)	14.2 (2.9)	60.9 (8.2)	10 (3.3)
No	20.1 (4.3)	13.4 (4.3)	12.5 (3.4)	14.8 (3.0)	58.1 (9.6)	10.2 (3.5)
*P* value	.12	<.001	<.001	.09	.008	.65
Previous knowledge about AI, mean (SD)						
Yes	20.5 (4.1)	15.4 (4.7)	13.2 (3.6)	14.6 (2.8)	60.6 (8.2)	10.1 (3.4)
No	20.2 (4.7)	13 (4.4)	13.7 (4.1)	14.5 (3.3)	55.8 (10.6)	10.2 (3.6)
*P* value	.59	<.001	.32	.95	<.001	.84

a GPA: grade point average.

Increased self-efficacy with computers was correlated with decreased barriers to accessing AI technology, lower computer anxiety scale scores (*r*=−0.27, *P*<.001 and *r*=−-0.568, *P*<.001, respectively), and an increase in the perception of using AI *(r*=0.27, *P*<.001). Meanwhile, nursing students’ readiness to adopt AI in nursing practice was negatively associated with barriers to accessing AI technology (*r*=−0.20, *P*<.001) and positively associated with attitudes toward and perceptions of using AI (*r*=0.32, *P*<.001 and *r*=0.14, *P*=.01, respectively). An increase in barriers to accessing AI technology was associated with a negative attitude toward AI and the perception of using AI by nursing students (*r*=−0.34, *P*<.001 and *r*=−0.39, *P*<.001, respectively), as shown in Table 3.

**Table 3.** The correlation among nursing internship students’ readiness, self-efficacy, barriers, attitudes, perceptions, and anxiety to use AI.

Variable and correlations	Readiness	Self-efficacy	Barriers	Attitude	Perception	Anxiety
Readiness						
* r*	1	0.103	−0.200	0.321	0.144	−0.031
*P* value	—^a^	.07	<.001	<.001	.01	.59
Self-efficacy						
*r*	0.103	1	−0.270	0.118	0.269	−0.568
*P* value	.07	—	<.001	.04	<.001	<.001
Barriers						
*r*	−0.200^b^	−0.270^b^	1	−0.336	−0.387	0.111
*P* value	<.001	<.001	—	<.001	<.001	.05
Attitude						
*r*	0.321	0.118	−0.336^b^	1	0.196	−0.041
*P* value	<.001	.04	<.001	—	<.001	.48
Perception						
*r*	0.144^c^	0.269^b^	−0.387^b^	0.196^b^	1	−0.087
*P* value	.01	<.001	<.001	<.001	—	.13
Anxiety						
*r*	−0.031	−0.568^b^	0.111	−0.041	−0.087	1
*P* value	.59	<.001	.05	.48	.13	—

a Not applicable.

b Correlation is significant at the .01 level (2-tailed).

c Correlation is significant at the .05 level (2-tailed).

### Predictors of Nursing Internship Students' Readiness to Use AI

**Figure 1.**
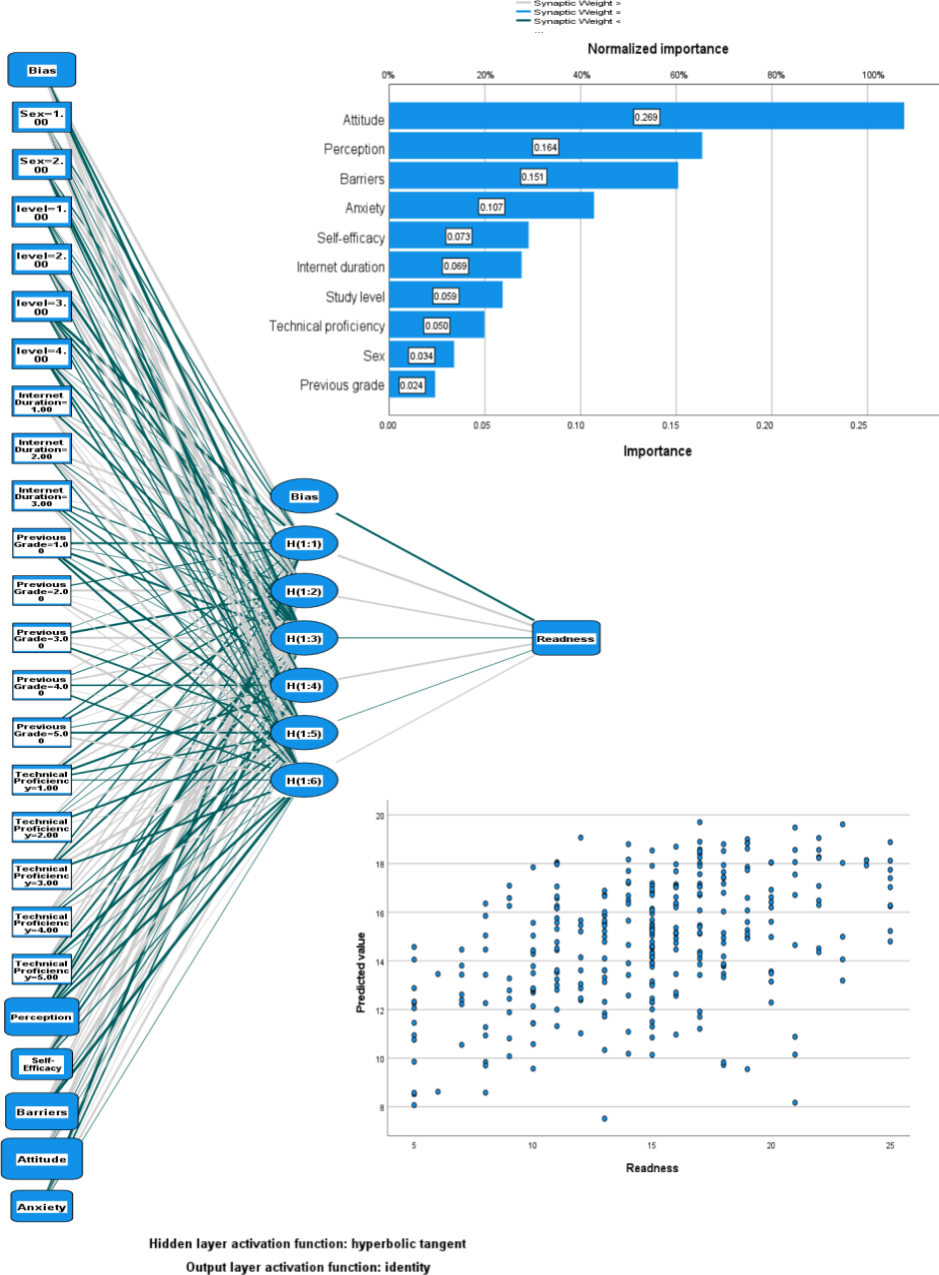
Factors affecting nursing internship students' readiness to use AI.

A multilayer neural network was used to predict nursing internship students’ readiness to adopt AI, based on specified independent factors. The model identified barriers (relative importance=0.27), attitudes (relative importance=0.16), and perceptions (relative importance=0.15) as the strongest predictors of readiness. Self-efficacy (relative importance=0.11) and anxiety (relative importance=0.07) demonstrated comparatively smaller contributions despite their nonsignificant bivariate correlations with readiness. The findings shown in Figure 1 suggest that these factors collectively influenced nurses’ openness to AI integration. The model demonstrated strong predictive validity, minimized error profiles, and high stability. While the training SSE was 65.93, the model achieved a low training relative error of 0.62, indicating high explanatory adequacy. The generalization and predictive ability of the network were confirmed by a testing SSE of 35.49 and a low testing relative error, establishing that the model was structurally stable and free from overfitting constraints.

## Discussion

### Principal Findings

This study aimed to assess how nursing students’ attitudes, perceptions, self-efficacy, barriers, and anxiety influenced their readiness to adopt AI in nursing practice. The readiness of nursing students to incorporate AI in their practice is influenced by several factors, such as gender, technology skills, knowledge of the applicability of AI, and most importantly, knowledge background. Comprehensively addressing these factors through oriented teaching may equip nursing students with better ways to incorporate AI into their future practice and consequently enhance patient outcomes. Students aged over 20 years, studying at the fourth level, and having excellent GPAs in the last semester had higher mean self-efficacy with computers.

The readiness of nursing students to adopt AI in nursing practice was higher among male students, those with excellent technological proficiency, those aware of the use of AI in health care and nursing care, and those with prior knowledge of AI. Additionally, students with significant technological abilities tend to be more self-assured in their capacity to study and apply technologies such as AI, which has been clearly linked to their preparedness to embrace AI in nursing practice [31]. According to previous studies, male students are more likely to be ready to operate with AI than female students [31,32]. This is likely due to sociocultural factors that encourage males to take an interest in technological aspects and be less risk averse [32].

Nursing students’ perceptions of AI had a higher mean score among those who were aware of the use of AI in health care and nursing care. However, barriers to accessing AI technology were higher among students in the fourth level. This suggests that nursing students who are familiar with the application of AI in the health sector may tend to have favorable sentiments toward it. However, especially during the graduate year, fourth-year students may not be able to access and use AI technology for reasons such as graduation pressure, insufficient time, and resources. Students exposed to further uses of AI in a clinical environment develop a more favorable attitude toward AI [33]. Nevertheless, as many positive perceptions arise as a result of awareness, obstacles to the appreciation of AI technology are a real source of concern, mostly for fourth-year undergraduate nurses. Fourth-year students may experience increased academic and career-related pressures, which could contribute to perceived barriers to engaging in AI technologies. This is in line with those who argue that, due to the underdevelopment of certain components of nursing education programs, such as health informatics and AI incorporating education, feelings of inferiority may arise and inhibit students’ ability to use these technologies [34]. This educational gap may contribute to the barriers experienced by these students, as they may not feel adequately prepared to engage with AI technologies in clinical settings.

Nursing students’ attitudes toward AI were more positive among those with excellent technological proficiency, those aware of AI’s use in health care and nursing care, and those with prior knowledge of AI. Additionally, computer anxiety was higher among students aged 20 years or younger and among those with a fair GPA in the previous semester. This finding indicates that younger nursing students may experience greater apprehension when using computer technologies, highlighting the importance of early exposure to digital technologies and targeted educational support to enhance confidence and reduce technology-related anxiety. Owing to the increasing integration of AI worldwide, there is a dire need for nursing education that integrates AI knowledge to enhance the willingness of technology-based students [34]. It is also pertinent to note that awareness of the application of these technologies among nursing students also has an impact on their attitudes toward nursing. Previous studies have noted that students and professionals perceive AI to be more positivistic and feasible if they are aware of implications such as improved patient outcomes and decision-making [11,17]. Considering the prospects of AI in health care, it is essential to focus on educational activities that will allow future nurses to fully apply this knowledge in practice.

Meanwhile, nursing students’ readiness to adopt AI in nursing practice was associated with fewer barriers to accessing AI technology, a more positive attitude toward AI, and a more positive perception of AI use. Greater familiarity with AI implementation was associated with more favorable attitudes toward AI technology, suggesting that increased exposure to and experience with AI may enhance students’ acceptance of and confidence in using these technologies. These students were confirmed to be more positive about AI technologies and wanted to use them in their work [17,35]. From this, one can deduce that attention directed toward the progression of students’ understanding of AI leads to an increased possibility of supporting positive change.

In contrast, high-level barriers to entering AI technology correlate with negative attitudes toward and perceptions of AI. For instance, nursing students who do not have easy access to AI tools may develop negative feelings, such as anxiety and negligence toward these technologies, which could discourage them from using them. This was consistent with a study conducted by Chen et al [36] in which perceived barriers were shown to reduce students’ intent to engage in AI-related activities. In addition, this study highlights the need to overcome these barriers because students’ attitudes toward AI are important for the acceptance and subsequent use of AI-powered applications in nursing [37]. This study used neural network modeling to ascertain the participants’ AI adoption styles. There was a higher degree of accuracy in predicting readiness, with barriers, attitudes, perceptions, self-efficacy, and anxiety emerging as the top 5 manifest variables. In the case of readiness, there were also variables such as lack of access to AI technology, attitudes toward AI technology, beliefs about the usefulness of the technology, feelings of self-efficacy, and feelings of anxiety about the new technology. AI technology is positively and directly associated with perceptions of usefulness [20]. For example, it has been reported that nursing students’ perceptions of AI technologies predict more positive attitudes toward the use of AI in nursing practice [17]. Indeed, training and support remove barriers and improve self-efficacy, thereby increasing the propensity to use AI [38]. Obenza et al [39] supported the notion that self-efficacy is an important determinant of individuals’ willingness and confidence in adopting emerging technologies. There are various estimations among nursing students regarding the implementation of AI when providing nursing care, and these vary in the adoption of AI owing to their attitudes and concerning factors. For instance, high perceived barriers to using AI technology tend to correlate negatively with positive feelings toward AI and the perception of its usefulness. In cases where nursing students possess barriers such as poor orientation and deficit of resources or scanty institutional support, they are likely to develop a certain disbelief in the usage of these AI-based tools. In such cases, anxiety about these problems is more likely to arise, which, according to [17], is negatively related to self-efficacy and willingness to accept changes in practices, including new technologies [17] in a negative sense. However, because of this anxiety, there is a high chance that students will be reluctant to use AI, which will lead to a negative attitude toward the adoption of new technology, which is likely to result in a negative loop, as pointed out [40]. Therefore, it is essential to address students’ anxiety regarding AI to eliminate this negative loop and establish better interactions between nursing students and their modern technologies.

Despite the contribution of self-efficacy and anxiety to the neural network model, they did not correlate substantially with readiness in bivariate studies. This disparity may be explained by the capacity of neural networks to identify interaction effects and nonlinear interactions that are missed by straightforward correlations. Therefore, rather than being direct indicators of preparedness, their involvement should be carefully understood as indirect or context dependent. Self-efficacy is an important determinant of nursing students’ perceptions of AI. A recent study indicated that students with greater self-efficacy regarding AI tend to approach and use AI technologies more positively [39]. It has been noted that self-efficacy is an important variable that affects the use of any technology, suggesting that students with such self-efficacy will be able to break through barriers and embrace new technology optimization [41]. Therefore, if the root causes of low self-efficacy concerning AI use in nursing practice are addressed with appropriate educational measures, there is a high likelihood that nurses will accept and use this technology while performing their duties. When self-efficacy is improved through adequate training programs and AI barriers are uplifted, nursing teachers can positively alter students’ attitudes toward using AI. This approach also resonates with an earlier research suggestion that health care professionals should work together to facilitate effective integration of AI technologies into nursing practice [42]. Furthermore, the literature highlights the need to consider students’ perceptions and attitudes toward AI. Anxiety factors, such as fear of incomprehension and subordination, are negatively influenced by the intention to use AI in nursing students [17]. Filling students’ minds with information on the availability and advantages of AI technologies can increase their acceptance of and intention to use such tools. This is especially pertinent for nursing education, where incorporating AI into curricula could help students better comprehend it and decrease the fear associated with its application. However, the use of self-reported measures increases the risk of social desirability effects and common method bias, which could exaggerate the observed correlations between variables. The correlations may have been overstated because multiple categories (such as attitudes, self-efficacy, and perception) were measured at a single time point using the same methodology. The prediction performance of a neural network model may also be affected by inflation. Therefore, the strength of this relationship should be cautiously interpreted.

### Limitations

Despite revealing some important information, the limitations of this study include country restrictions, use of self-reporting methods, use of only survey instruments, a lack of emphasis on ethical and cultural dimensions, the absence of tools within the study that might be needed, absence of data regarding AI use by students, and consideration of only short-term issues. To mitigate or even eliminate such limitations, it is recommended that future studies cover more, use what is available, relate the variables, detail ethics and cultures, check the clients’ tools, look for students’ embedment in AI, and assess their anxiety at a distance. This helps to expand the knowledge of how nursing students perceive AI in their future practice and helps form clinically effective approaches to the integration of AI into nursing practice. Although the AI readiness scale was previously updated for nursing students, it was originally designed for different professional groups. In addition, the instruments used in this study were originally developed and validated in different cultural contexts. Although administered in English, the absence of formal cross-cultural validation in the study population may have affected the generalizability and cultural equivalence of the findings. Additionally, the use of a neural network model, which is advantageous for prediction, limits interpretability compared to traditional statistical methods, which may affect the explanatory depth of the findings. Moreover, this study relied solely on self-reported data, which are susceptible to frequent method bias and social desirability effects. These biases may result in inflated effect sizes and overestimation of correlations between variables, as well as potentially improve model prediction accuracy. To avoid these biases, future research should use objective metrics, multiple data sources, or longitudinal approaches.

### Implications for Nursing

Nursing students’ competence in using their technological knowledge and skills to adopt and implement AI in their careers is explained by their positive attitudes toward and understanding of the benefits of AI. However, factors such as anxiety and limited access can reduce competence. To solve these problems, it is necessary to embed AI in nursing education, provide practical experience, eliminate fear, change public perception, and highlight its benefits. Furthermore, AI education should address ethical and cultural issues explicitly. To guarantee ethical use, issues such as data privacy, algorithmic bias, patient safety, and cultural sensitivity in AI-supported decision-making are crucial. Educational practices should be context-sensitive and prioritize fairness and accessibility due to differences in access to technology and institutional resources. Such integration will prepare future nurses to accept AI in their practice to advance the level of care patients receive in the health care system.

### Conclusions

Several contributing factors influence nursing students’ readiness to embrace AI, with barriers, attitudes, and perceptions emerging as the most consistent factors, while self-efficacy and anxiety may play indirect roles. The receptive attitudes of students with more skills, awareness, computer proficiency, and positive beliefs have encouraged the use of AI. Conversely, barriers, particularly higher levels of anxiety among younger students (≤20 y) and those with a lower GPA, were associated with lower readiness to use AI. A neural network model affirms the effects of various factors in predicting readiness to embrace AI. Creating a supportive educational environment that fosters AI readiness, self-efficacy, and positive attitudes toward AI may facilitate appropriate integration of AI into nursing education and future clinical practice.

## Abbreviations

GPA: grade point average
SSE: sum of squares error
